# Changes in Tobacco Product Use Among Students Aged 13 to 15 Years in 34 Countries, Global Youth Tobacco Survey, 2012–2020

**DOI:** 10.5888/pcd20.220410

**Published:** 2023-08-03

**Authors:** Gibril J. Njie, Candace Kirksey Jones, Nerline Jacques, Adewole Adetokun, James Ross, Ashlyn Owens, Linda Anton, Michelle Johns, Liping Pan

**Affiliations:** 1 Global Tobacco Control Branch, Office on Smoking and Health, National Center for Chronic Disease Prevention and Health Promotion, Centers for Disease Control and Prevention, Atlanta, Georgia; 2 RTI International, Research Triangle Park, North Carolina; 3 CyberData Technologies, Herndon, Virginia; 4 Oak Ridge Institute for Science and Education, Oak Ridge, Tennessee; 5 Chenega Enterprise Systems and Solutions, LLC, Chesapeake, Virginia

## Abstract

**Introduction:**

Most adults who currently use tobacco start before age 21. Comprehensive, cost-effective strategies and interventions to prevent initiation and encourage tobacco use cessation among youth are critical aspects of protecting youth from the harms of commercial tobacco. We describe changes in current tobacco product use among youth in 34 sites using data from the Global Youth Tobacco Survey (GYTS).

**Methods:**

GYTS is a nationally representative school-based survey of students aged 13 to 15 years. The analysis included 34 sites that completed 2 survey waves during 2012–2020. Prevalence of current tobacco use was assessed for each country. Marginal effects in multivariable logistic regression models were used to estimate adjusted prevalence difference (aPD) between waves.

**Results:**

The adjusted prevalence of current tobacco product use remained unchanged in more than 60% of the included sites. For any tobacco use, significant decreases were reported for Bhutan (aPD = −8.1; 95% CI, −12.9 to −3.4), Micronesia (aPD = −7.2; 95% CI, −9.7 to −4.7), San Marino (aPD = −7.0; 95% CI, −11.2 to −2.7), Togo (aPD = −2.7; 95% CI, −4.6 to −0.7), and Panama (aPD = −2.2; 95% CI, −4.1 to −0.4); significant increases were reported for Moldova, Albania, and Paraguay. Current e-cigarette use increased significantly in 7 of 10 sites.

**Conclusion:**

Data show that progress toward reducing tobacco use among youth stalled during 2012–2020, while e-cigarette use increased in a few sites with available data.

**Summary**

**What is already known on the topic?**

Tobacco use is a leading cause of disease and death globally; most adults who currently use tobacco started before age 21 years.

**What is added by this report?**

During 2012–2020, self-reported current tobacco use remained unchanged between survey waves in more than 60% of the countries that implemented the Global Youth Tobacco Survey, while e-cigarette use increased in most of the countries with comparison data.

**What are the implications for public health practice?**

Low- and middle-income countries might consider adopting, implementing, and enforcing comprehensive tobacco control policies, such as those outlined in the World Health Organization MPOWER package, to reduce the availability and accessibility of tobacco products among youth.

## Introduction

Tobacco use is a leading cause of preventable death and disease worldwide. In 2019, an estimated 1 billion people globally used tobacco regularly, with nearly 8 million attributable deaths (1). Tobacco use often starts during adolescence, with most adults who currently smoke tobacco having initiated before age 21 (2). Although cigarettes are the most common tobacco product used by youth globally, the product landscape continues to expand, including various combustible, noncombustible, and electronic products (3,4).

Data from 2010–2020 indicate the average global prevalence of current use of any tobacco product among youth aged 13 to 15 years was 10.3%, with smokeless tobacco use at 2.6% and cigarette smoking at 6.0% (4). Globally, the prevalence of current cigarette smoking among youth has declined over the past 30 years (2,5). Despite this decline in cigarette smoking, the prevalence of tobacco use remains relatively high among youth in low- and middle-income countries (LMICs) (2). The World Health Assembly adopted the World Health Organization (WHO) Framework Convention on Tobacco Control (FCTC) in 2003. The WHO FCTC — an international treaty for public health — came into force in 2005 and included measures to guide the adoption and implementation of evidence-based interventions and strategies to reduce tobacco demand. As of May 2020, 182 Parties, covering 90% of the world’s population, had ratified the treaty (6,7). Article 20 of the WHO FCTC calls for Parties to establish a tobacco surveillance system to monitor indicators related to tobacco consumption. Among Parties to the WHO FCTC, the Global Youth Tobacco Survey (GYTS) is the most used surveillance system to monitor youth tobacco consumption (8).

The GYTS standard protocol was updated by WHO and the Centers for Disease Control and Prevention (CDC) in 2012 to ensure consistency in tobacco product use definitions across countries. However, few studies have considered the updates in the standard protocol when analyzing GYTS data. We assessed changes in the country-specific prevalence of current (ie, past 30-day) tobacco product use among students aged 13 to 15 years from 34 countries and sites (hereinafter “sites”) that have conducted at least 2 waves of GYTS since 2012.

## Methods

### Study sample

The GYTS is a cross-sectional, nationally representative, school-based survey of students in grades primarily associated with ages 13 to 15 years (9). GYTS uses a 2-stage cluster sampling design; the first stage consists of sampling schools based on the probability of selection proportional to school enrollment size, and the second stage consists of randomly selecting eligible classes within the selected schools. The GYTS is paper-based, anonymous, and self-administered, and all students in the selected classes are eligible to participate. Details on the survey methods, instrument, and public use data sets are published elsewhere (10,11).

We analyzed GYTS data from 34 sites that implemented at least 2 waves between 2012 and 2020. These sites were Albania, Argentina, Bhutan, Federation of Bosnia and Herzegovina, Brunei Darussalam, Federated States of Micronesia, Gaza Strip/West Bank, Georgia, Guam, Indonesia, Iraq, Italy, Kyrgyzstan, Latvia, Lithuania, Moldova, Mongolia, Montenegro, Nicaragua, Palau, Panama, Paraguay, Peru, Philippines, Qatar, Republic of Srpska, Romania, San Marino, Senegal, Serbia, Tajikistan, Timor-Leste, Togo, and Uruguay. The study included 102,011 and 105,024 students aged 13 to 15 years for the base and latest waves, respectively. Sample size for the base wave ranged from 534 to 6,178 students for San Marino and Mongolia, respectively; the latest wave ranged from 544 to 6,670 students for San Marino and the Philippines, respectively. The response rate for the base wave ranged from 57.4% in Micronesia to 100% in Palau; the response rate for the latest wave ranged from 52.2% in Serbia to 95.3% in the West Bank. All 34 sites included in the analysis reported comparison data for current cigarette smoking. Thirty-three (97%) sites reported comparison data for smoking tobacco products other than cigarettes and using any tobacco product; 32 (94%) sites reported comparison data for smokeless tobacco, and 10 (29%) sites reported comparison data for electronic (e-)cigarettes. Thirty (88%) of the 34 sites conducted their base wave in 2013 (14 sites) or 2014 (16 sites); 20 sites (59%) conducted their latest wave in 2019. We considered the base wave to be the first wave immediately following implementation of the 2012 standard protocol.

This study was not subject to human subjects’ review as a secondary analysis of deidentified public-use data. Data analyses were completed in September 2022.

### Measures

The primary outcomes of this analysis were the changes in current use of cigarettes, smoked tobacco products other than cigarettes, smokeless tobacco, any tobacco product use, and electronic cigarettes for each country. The definitions for tobacco use were as follows:

- Current cigarette use was defined as students answering 1 or more to the question “During the past 30 days, on how many days did you smoke cigarettes?” Response categories were 0, 1 or 2, 3–5, 6–9, 10–19, 20–29, or all 30 days.
- Current use of smoked tobacco products other than cigarettes was defined as students answering yes to the question, “During the past 30 days, did you use any form of smoked tobacco products other than cigarettes (such as cigars, pipes, waterpipes/shisha, bidis)?” Response categories were yes or no.
- Current use of smokeless tobacco was defined as students answering yes to the question, “During the past 30 days, did you use any form of smokeless tobacco products (such as snuff, chewing tobacco, dip, betel quid with tobacco)?” Response categories were yes or no.
- Current use of any tobacco product was defined as students answering 1 or more days for cigarette use in the past 30 days, or yes for the current use of smoked tobacco products other than cigarettes in the past 30 days, or yes for using smokeless tobacco in the past 30 days.
- Current use of electronic cigarettes was defined as students answering 1 or more to the questions, “During the past 30 days, on how many days did you use electronic cigarettes?” and “During the last 30 days, did you use electronic cigarettes?” Response categories were 0, 1 or 2, 3–5, 6–9, 10–19, 20–29, or all 30 days. Response categories were yes or no.

We also included as covariates sex (“What is your sex?” with response categories as male or female), age (“How old are you?” with response categories as 11, 12, 13, 14, 15, 16, 17 years old), grade (“In what grade/form are you?” with responses varied by country based on education system), year of survey (varied by country), and the presence of a person who smokes in the household, which was defined as students answering 1 or more days to the question “During the past 7 days, on how many days has anyone smoked inside your home, in your presence?” Response categories were 0 days, 1 to 2 days, 3 to 4 days, 5 to 6 days, and 7 days.

### Statistical analysis

We computed weighted crude prevalence and 95% CIs for youth reporting current use in each site for the base and latest waves for the following products: cigarettes, smoked tobacco products other than cigarettes, smokeless tobacco products, any tobacco product, and electronic cigarettes. Prevalence estimates and 95% CIs were suppressed if the total unweighted sample size was less than 35. Marginal effects in multivariable logistic regression models were obtained to represent each site’s adjusted prevalence estimate for each wave, controlling for the covariates. Multivariable logistic regression models were also used to compute the adjusted prevalence difference (aPD) for each site between survey waves; the adjusted prevalence differences were considered significant if the 2-sided *P* value was less than .05. Analyses were conducted using SAS version 9.4 (SAS Institute Inc) and SAS-callable SUDAAN version 11.0.3 (RTI International) to account for the complex sampling design as well as school and student nonresponse.

## Results

### Current cigarette smoking

Adjusted prevalence estimates for the base wave ranged from 0.6% (95% CI, 0.4% to 1.2%) for Tajikistan to 29.9% (95% CI, 27.4% to 32.6%) for Palau; the latest wave ranged from 0.7% (95% CI, 0.3% to 1.5%) to 35.0% (95% CI, 30.4% to 39.8%) for Tajikistan and Palau, respectively (Table 1). Between the base and the latest waves, 4 sites — Micronesia, Qatar, San Marino, and Timor-Leste — had significant decreases in the prevalence of current cigarette smoking among youth aged 13 to 15 years. The aPD ranged from −2.8 percentage points (95% CI, −5.3 to −0.3) for Qatar to −7.4 percentage points (95% CI, −13.9 to −1.0) for Timor-Leste. One country, Palau, had a significant increase in youth cigarette smoking, with an aPD of 5.0 percentage points (95% CI, 0.7 to 9.3). The remaining 29 sites (85%) had no significant change in the prevalence of youth who reported smoking cigarettes on 1 or more days in the past 30 days.

**Table 1:** Changes in the Prevalence of Cigarette Smoking Among Youth Aged 13–15 Years, by Country, Global Youth Tobacco Survey, 2012–2020

Country or site	Crude prevalence		Adjusted prevalence^a^		Adjusted prevalence difference, pct pts (95% CI)
	Base wave, % (95% CI)	Latest wave, % (95% CI)	Base wave, % (95% CI)	Latest wave, % (95% CI)	
Argentina	19.6 (16.3 to 23.3)	17.8 (10.8 to 28.0)	17.0 (14.3 to 20.0)	19.8 (12.8 to 29.4)	2.9 (−5.3 to 11.0)
Panama	4.9 (4.2 to 5.8)	3.9 (2.9 to 5.2)	4.9 (4.5 to 5.8)	3.9 (2.9 to 5.2)	−1.1 (−2.5 to 0.4)
Bhutan	14.0 (11.8 to 16.4)	14.7 (12.9 to 16.7)	14.7 (12.4 to 17.4)	14.6 (12.9 to 16.4)	−0.2 (−3.1 to 2.8)
Bosnia and Herzegovina	18.1 (15.2 to 21.3)	13.7 (11.6 to 16.2)	17.3 (14.5 to 20.6)	14.4 (11.1 to 18.5)	−2.9 (−8.3 to 2.6)
Brunei	8.5 (5.1 to 13.9)	6.1 (4.5 to 8.1)	8.3 (5.5 to 12.4)	6.0 (4.5 to 8.0)	−2.3 (−5.7 to 1.1)
Gaza Strip/West Bank	6.4 (4.3 to 9.5)	6.9 (4.2 to 11.1)	6.2 (4.7 to 8.1)	6.5 (4.2 to 9.9)	0.3 (−2.3 to 2.8)
Micronesia	27.3 (27.3 to 27.3)	20.6 (18.7 to 22.6)	25.2 (23.7 to 26.8)	21.8 (19.4 to 24.4)	−3.5 (−5.8 to −1.2)^b^
Moldova	7.2 (5.2 to 9.8)	7.4 (6.0 to 9.1)	7.2 (5.1 to 9.9)	7.3 (6.1 to 8.8)	0.1 (−2.7 to 3.0)
Palau	32.1 (32.1 to 32.1)	32.6 (28.2 to 37.2)	29.9 (27.4 to 32.6)	35.0 (30.4 to 39.8)	5.0 (0.7 to 9.3)^b^
Qatar	9.6 (6.5 to 13.9)	6.6 (4.8 to 9.0)	9.3 (7.0 to 12.1)	6.5 (4.8 to 8.7)	−2.8 (−5.3 to −0.3)^b^
Republic of Srpska	7.8 (5.6 to 10.8)	9.2 (6.9 to 12.0)	7.1 (5.1 to 9.8)	10.6 (7.4 to 14.9)	3.5 (−0.9 to 7.9)
Romania	9.3 (7.7 to 11.3)	8.6 (7.3 to 10.1)	9.9 (8.3 to 11.8)	7.9 (6.7 to 9.3)	−2.0 (−4.1 to 0.1)
Senegal	3.9 (2.12 to 7.14)	3.3 (2.4 to 4.5)	3.6 (2.06 to 6.13)	3.7 (2.5 to 5.4)	0.1 (−2.2 to 2.4)
Serbia	13.0 (10.4 to 16.0)	11.0 (11.0 to 11.0)	12.9 (10.4 to 15.9)	11.6 (10.3 to 13.0)	−1.3 (−3.5 to 0.9)
Timor-Leste	28.8 (22.0 to 36.8)	20.1 (16.1 to 24.9)	28.1 (22.6 to 34.3)	20.6 (16.7 to 25.3)	−7.4 (−13.9 to −1.0)^b^
Togo	4.8 (3.5 to 6.5)	2.8 (1.9 to 4.1)	4.1 (3.0 to 5.5)	3.2 (2.3 to 4.5)	−0.9 (−2.5 to 0.7)
Georgia	6.9 (4.3 to 10.9)	8.4 (6.1 to 11.6)	6.8 (4.3 to 10.9)	8.4 (6.3 to 11.1)	1.6 (−2.2 to 5.4)
Guam	14.9 (11.7 to 19.0)	10.6 (8.5 to 13.1)	12.1 (10.3 to 14.3)	12.1 (10.3 to 14.3)	0 (NA)
Indonesia	18.2 (13.9 to 23.5)	19.2 (16.8 to 21.8)	17.8 (14.2 to 22.0)	19.6 (17.3 to 22.1)	1.8 (−2.0 to 5.6)
Iraq	5.7 (3.7 to 8.7)	8.2 (5.0 to 13.0)	5.8 (4.0 to 8.4)	7.7 (5.1 to 11.4)	1.8 (−1.0 to 4.7)
Italy	23.5 (20.8 to 26.8)	19.8 (17.1 to 22.9)	20.3 (17.0 to 24.2)	19.7 (17.0 to 22.7)	−0.7 (−4.4 to 3.1)
Kyrgyzstan	2.4 (1.6 to 3.5)	2.4 (1.4 to 4.1)	2.5 (1.7 to 3.8)	2.2 (1.3 to 3.6)	−0.3 (−1.7 to 1.1)
Latvia	16.7 (15.1 to 18.5)	14.8 (12.1 to 17.9)	16.2 (14.7 to 17.8)	15.2 (12.7 to 18.1)	−1.0 (−3.9 to 1.9)
Lithuania	19.5 (17.0 to 22.3)	16.7 (14.0 to 20.0)	19.1 (16.8 to 21.7)	15.7 (12.7 to 19.3)	−3.4 (−7.7 to 1.0)
Mongolia	3.9 (3.1 to 4.8)	4.8 (3.7 to 6.3)	3.9 (3.2 to 4.7)	4.6 (3.5 to 6.0)	0.7 (−0.6 to 2.1)
Montenegro	6.9 (3.4 to 13.5)	6.0 (4.9 to 7.3)	6.1 (3.3 to 11.1)	6.7 (5.0 to 9.0)	0.6 (−2.1 to 3.3)
Nicaragua	12.0 (10.4 to 13.9)	10.8 (9.8 to 12.5)	10.7 (9.2 to 12.5)	11.5 (10.0 to 13.2)	0.7 (−1.5 to 3.0)
Paraguay	3.9 (3.2 to 4.6)	3.0 (2.5 to 3.6)	3.6 (3.0 to 4.4)	3.1 (2.5 to 3.7)	−0.5 (−1.5 to 0.4)
Peru	7.7 (5.3 to 11.1)	4.9 (3.5 to 6.9)	7.2 (5.3 to 9.8)	5.2 (3.8 to 7.1)	−2.0 (−4.4 to 0.3)
San Marino	12.9 (0.8 to 16.9)	6.0 (3.9 to 8.9)	12.8 (9.9 to 16.6)	6.0 (4.0 to 8.8)	−6.8 (−10.8 to −2.9)^b^
Tajikistan	0.8 (0.4 to 1.4)	0.7 (0.3 to 1.5)	0.6 (0.4 to 1.2)	0.7 (0.3 to 1.5)	0 (−0.6 to 0.6)
Uruguay	8.0 (6.5 to 9.8)	8.6 (5.1 to 14.1)	8.1 (6.5 to 10.0)	8.4 (5.0 to 13.7)	0.3 (−4.4 to 4.9)
Albania	6.0 (4.8 to 7.6)	4.4 (3.7 to 5.3)	6.0 (4.6 to 7.6)	4.1 (3.4 to 5.0)	−1.8 (−3.7 to 0.0)
Philippines	12.0 (10.0 to 14.2)	10.0 (8.1 to 12.3)	11.4 (9.4 to 13.6)	10.3 (8.4 to 12.5)	−1.1 (−4.0 to 1.8)

Abbreviations: NA, not applicable; pct pts, percentage points.

a Adjusted for sex, age, grade, survey year, and presence of a person who smokes in the household.

b Significant at *P* < .05 in multivariable logistic regression model.

### Current other smoked tobacco not including cigarettes

Table 2 shows the change in the prevalence of youth who reported smoking tobacco products other than cigarettes on 1 or more days during the past 30 days. Adjusted base wave prevalence estimates ranged from 1.6% (95% CI, 1.2% to 2.3%) to 17.5% (95% CI, 15.9% to 19.3%) for Moldova and Palau, respectively; latest wave estimates ranged from 1.6% (95% CI, 1.2% to 2.3%) in Tajikistan to 22.3% (95% CI, 18.4% to 26.7%) for Bosnia and Herzegovina. Of the 33 sites that collected data for this indicator, 3 sites (9.0%) — Panama (aPD = −2.0; 95% CI, −3.0 to −0.9), Micronesia (aPD = −2.1; 95% CI, −3.6 to −0.6), and Palau (aPD = −5.5; 95% CI, −8.1 to −2.9 — had significant decreases in the prevalence of youth who reported smoking other tobacco products in the past 30 days. Ten sites (30%) — Bhutan, Bosnia and Herzegovina, Gaza Strip and West Bank, Moldova, Iraq, Latvia, Mongolia, Montenegro, Paraguay, and Albania — had significant increases in the change in prevalence of smoking other tobacco products in the past 30 days. The magnitude of prevalence change ranged from 1.1 percentage points (95% CI, 0.2 to 2.0) for Mongolia to 12.3 percentage points (95% CI, 7.4 to 17.2) for Bosnia and Herzegovina. Most sites (61%) did not report a significant change in the prevalence of youth smoking tobacco products other than cigarettes.

**Table 2:** Change in Prevalence of Smoking Other Tobacco Products Not Including Cigarettes Among Youth Aged 13–15 Years, by Country, Global Youth Tobacco Survey, 2012–2020

Country or site	Crude prevalence		Adjusted prevalence^a^		Adjusted prevalence difference, pct pts (95% CI)
	Base wave, % (95% CI)	Latest wave, % (95% CI)	Base wave, % (95% CI)	Latest wave, % (95% CI)	
Argentina	6.5 (5.0 to 8.3)	4.3 (2.9 to 6.4)	5.7 (4.4 to 7.4)	5.0 (3.6 to 6.9)	−0.7 (−2.8 to 1.4)
Panama	4.3 (3.6 to 5.1)	2.4 (1.8 to 3.3)	4.4 (3.6 to 5.3)	2.4 (1.8 to 3.2)	−2.0 (−3.0 to −0.9)^b^
Bhutan	5.5 (4.2 to 7.0)	9.0 (7.8 to 10.4)	5.8 (4.5 to 7.4)	8.9 (7.8 to 10.2)	3.2 (1.2 to 5.1)^b^
Bosnia and Herzegovina	10.7 (8.9 to 13.2)	20.5 (17.8 to 23.5)	10.0 (8.1 to 12.3)	22.3 (18.4 to 26.7)	12.3 (7.4 to 17.2)^b^
Brunei	3.0 (1.9 to 4.9)	3.3 (2.3 to 4.6)	3.0 (2.0 to 4.6)	3.2 (2.2 to 4.5)	0.1 (−1.5 to 1.7)
Gaza Strip/West Bank	9.0 (6.6 to 12.0)	12.8 (8.6 to 18.7)	8.5 (6.6 to 10.9)	12.6 (9.1 to 17.0)	4.0 (0.3 to 7.8)^b^
Micronesia	12.8 (12.8 to 12.8)	9.6 (8.4 to 10.8)	12.0 (11.1 to 13.0)	9.9 (8.7 to 11.3)	−2.1 (−3.6 to −0.6)^b^
Moldova	1.6 (1.2 to 2.2)	9.5 (7.7 to 11.6)	1.6 (1.2 to 2.3)	9.4 (7.6 to 11.4)	7.7 (5.7 to 9.7)^b^
Palau	18.5 (18.5 to 18.5)	10.5 (8.7 to 12.6)	17.5 (15.9 to 19.3)	12.1 (10.1 to 14.4)	−5.5 (−8.1 to −2.9)^b^
Qatar	6.8 (4.6 to 9.9)	8.4 (6.5 to 10.9)	6.6 (4.8 to 9.0)	8.3 (6.6 to 10.4)	1.7 (−0.6 to 4.0)
Republic of Srpska	2.9 (2.4 to 3.5)	4.4 (3.7 to 5.2)	3.5 (3.1 to 4.0)	3.5 (3.1 to 4.0)	0 (NA)
Romania	3.7 (2.7 to 5.1)	6.19 (3.6 to 10.4)	3.8 (2.8 to 5.2)	6.1 (3.5 to 10.2)	2.3 (−1.2 to 5.7)
Senegal	4.9 (2.8 to 8.3)	4.9 (4.0 to 6.0)	4.2 (2.6 to 6.6)	5.8 (4.3 to 7.6)	1.6 (−0.6 to 3.7)
Serbia	3.9 (3.1 to 4.5)	7.08 (7.08 to 7.08)	4.0 (3.2 to 5.0)	4.0 (3.2 to 5.0)	0 (NA)
Timor-Leste	9.9 (7.5 to 12.9)	8.2 (6.3 to 10.6)	9.8 (7.8 to 12.3)	8.1 (6.2 to 10.6)	−1.7 (−4.8 to 1.4)
Togo	3.1 (2.4 to 4.1)	1.6 (1.0 to 2.3)	2.8 (2.1 to 3.7)	1.7 (1.1 to 2.6)	−1.0 (−2.1 to 0.0)
Georgia	4.1 (2.5 to 6.9)	6.1 (4.7 to 8.0)	4.0 (2.4 to 6.5)	6.2 (4.8 to 7.9)	2.2 (−0.2 to 4.5)
Guam	12.8 (10.2 to 16.1)	9.3 (7.6 to 11.4)	16.0 (9.8 to 25.0)	8.5 (6.2 to 11.6)	−7.5 (−17.0 to 2.0)
Indonesia	3.8 (2.8 to 5.0)	2.9 (2.3 to 3.5)	3.8 (3.0 to 4.9)	3.0 (2.4 to 3.7)	−0.8 (−1.9 to 0.2)
Iraq	7.5 (5.0 to 11.0)	12.3 (8.6 to 17.4)	7.7 (5.6 to 10.5)	11.6 (8.5 to 15.7)	3.9 (0.4 to 7.5)^b^
Kyrgyzstan	1.8 (1.1 to 2.8)	2.8 (2.1 to 3.8)	1.7 (1.1 to 2.7)	2.9 (2.1 to 3.8)	1.1 (0.0 to 2.2)
Latvia	12.0 (10.9 to 13.3)	14.1 (12.7 to 15.7)	11.8 (10.6 to 13.0)	14.4 (13.1 to 15.9)	2.7 (0.9 to 4.5)^b^
Lithuania	12.5 (10.4 to 14.9)	10.0 (8.2 to 12.2)	12.3 (10.4 to 14.5)	9.3 (7.2 to 11.9)	−3.0 (−6.4 to 0.3)
Mongolia	3.5 (2.8 to 4.3)	4.4 (3.8 to 5.0)	3.4 (2.8 to 4.1)	4.5 (3.8 to 5.2)	1.1 (0.2 to 2.0)^b^
Montenegro	2.6 (1.9 to 3.5)	4.4 (3.4 to 5.7)	2.4 (1.8 to 3.1)	4.4 (3.4 to 5.6)	2.0 (0.8 to 3.2)^b^
Nicaragua	4.4 (3.2 to 6.1)	4.3 (3.6 to 5.2)	4.3 (3.1 to 5.8)	4.4 (3.7 to 5.3)	0.2 (−1.3 to 1.7)
Paraguay	2.7 (1.8 to 3.9)	5.6 (4.2 to 7.5)	2.5 (1.7 to 3.6)	5.7 (4.2 to 7.6)	3.2 (1.3 to 5.1)^b^
Peru	3.8 (2.2 to 6.6)	2.9 (2.0 to 4.1)	3.5 (2.2 to 5.6)	3.1 (2.2 to 4.3)	−0.4 (−2.1 to 1.2)
San Marino	3.0 (1.5 to 5.8)	1.2 (0.6 to 2.6)	4.5 (2.7 to 7.4)	2.6 (1.6 to 4.3)	−1.8 (−4.7 to 1.1)
Tajikistan	1.7 (1.2 to 2.4)	1.2 (0.9 to 1.8)	1.8 (1.3 to 2.7)	1.6 (1.2 to 2.3)	−0.2 (−1.1 to 0.7)
Uruguay	2.7 (2.2 to 3.2)	3.2 (2.3 to 4.6)	2.9 (2.4 to 3.5)	3.0 (2.1 to 4.3)	0.1 (−1.1 to 1.3)
Albania	5.1 (4.3 to 6.1)	12.9 (10.9 to 15.0)	5.2 (4.3 to 6.3)	12.1 (10.3 to 14.3)	6.9 (4.6 to 9.2)^b^
Philippines	3.4 (2.0 to 5.6)	2.1 (1.7 to 2.7)	3.1 (1.9 to 4.9)	2.3 (1.8 to 2.9)	−0.8 (−2.3 to 0.7)

Abbreviations: NA, not applicable; pct pts, percentage points.

a Adjusted for sex, age, grade, survey year, and presence of a person who smokes in the household. “Other tobacco products” include cigars, pipes, waterpipes/shisha, or bidis.

b Significant at *P* < .05 in multivariable logistic regression model.

### Current smokeless tobacco use

The adjusted prevalence estimates for the base wave ranged from 1.0% (95% CI, 0.5% to 1.7%) to 23.1% (95% CI, 21.6% to 24.6%) for Brunei and Micronesia, respectively (Table 3). For the latest wave, adjusted prevalence estimates ranged from 0.8% (95% CI, 0.4% to 1.4%) to 16.6% (95% CI, 14.8% to 18.6%) for Togo and Micronesia, respectively. Nine sites (28%) — Argentina, Bhutan, Bosnia and Herzegovina, Micronesia, Republic of Srpska, Togo, Indonesia, Kyrgyzstan, and Uruguay — had significant decreases in the prevalence of smokeless tobacco use in the past 30 days among youth. Among these sites, Bhutan had the largest reduction for adjusted prevalence of smokeless tobacco use from its base wave in 2013 to its latest wave in 2019 at −10.3 percentage points (95% CI, −14.0 to −6.5); conversely, Indonesia had the smallest reduction in aPD at −1.0 (95% CI, −1.8 to −0.2) percentage points. Latvia and Montenegro were the only sites to report an increased aPD for smokeless tobacco use among youth who reported using on 1 or more days during the past 30 days. Like the outcomes for cigarette smoking and smoking of other tobacco products, most sites (66%) did not show a significant change in the use of smokeless tobacco products during the past 30 days.

**Table 3:** Change in Prevalence of Smokeless Tobacco Use Among Youth Aged 13–15 Years, by Country, Global Youth Tobacco Survey, 2012–2020

Country or site	Crude prevalence		Adjusted prevalence^a^		Adjusted prevalence difference, pct pts (95% CI)
	Base wave, % (95% CI)	Latest wave, % (95% CI)	Base wave, % (95% CI)	Latest wave, % (95% CI)	
Argentina	3.7 (2.9 to 4.8)	1.5 (0.8 to 2.9)	3.5 (2.7 to 4.5)	1.6 (0.9 to 2.8)	−1.9 (−3.1 to −0.7)^b^
Panama	2.8 (2.2 to 3.5)	2.3 (1.7 to 3.2)	2.8 (2.2 to 3.6)	2.2 (1.6 to 3.1)	−0.6 (−1.6 to 0.4)
Bhutan	21.6 (18.5 to 25.2)	12.5 (11.0 to 14.1)	22.7 (19.3 to 26.5)	12.5 (11.1 to 14.0)	−10.3 (−14.0 to −6.5)^b^
Bosnia and Herzegovina	3.1 (2.3 to 4.1)	1.7 (1.3 to 2.3)	4.8 (3.2 to 7.3)	1.1 (0.7 to 1.7)	−3.7 (−6.0 to −1.5)^b^
Brunei	0.9 (0.5 to 1.7)	1.2 (0.7 to 2.2)	1.0 (0.5 to 1.7)	1.3 (0.7 to 2.4)	0.3 (−0.7 to 1.4)
Gaza Strip/West Bank	5.4 (3.3 to 8.8)	4.3 (2.3 to 7.9)	5.3 (3.5 to 8.1)	4.3 (2.3 to 7.7)	−1.0 (−4.4 to 2.3)
Micronesia	23.8 (23.8 to 23.8)	16.0 (14.4 to 17.8)	23.1 (21.6 to 24.6)	16.6 (14.8 to 18.6)	−6.4 (−8.8 to −4.1)^b^
Moldova	2.2 (1.7 to 2.9)	1.7 (1.2 to 2.4)	2.4 (1.8 to 3.0)	1.5 (1.1 to 2.3)	−0.8 (−1.7 to 0.0)
Palau	19.3 (19.3 to 19.3)	14.5 (11.6 to 18.1)	18.8 (17.5 to 20.2)	15.1 (11.9 to 19.0)	−3.7 (−7.8 to 0.3)
Qatar	6.1 (4.5 to 8.2)	4.5 (3.0 to 6.7)	6.1 (4.6 to 7.9)	4.2 (2.6 to 6.1)	−1.9 (−4.1 to 0.3)
Republic of Srpska	1.6 (1.2 to 2.0)	1.7 (1.2 to 2.2)	2.5 (1.7 to 3.7)	1.1 (0.7 to 1.5)	−1.4 (−2.6 to −0.2)^b^
Senegal	4.4 (2.6 to 7.2)	3.5 (2.2 to 5.5)	3.9 (2.4 to 6.2)	4.2 (2.6 to 6.9)	0.4 (−2.1 to 2.9)
Serbia	1.6 (1.2 to 2.1)	1.8 (1.8 to 1.8)	1.6 (1.2 to 2.1)	1.6 (1.2 to 2.1)	0 (NA)
Timor-Leste	8.4 (6.6 to 10.7)	13.7 (11.2 to 16.5)	9.1 (7.1 to 11.4)	12.6 (10.6 to 15.1)	3.6 (0.5 to 6.6)
Togo	2.1 (1.5 to 3.0)	0.7 (0.4 to 1.3)	2.0 (1.4 to 2.8)	0.8 (0.4 to 1.4)	−1.2 (−2.0 to −0.4)^b^
Georgia	3.4 (2.6 to 4.5)	4.1 (2.2 to 7.5)	3.4 (2.4 to 4.7)	4.1 (2.2 to 7.4)	0.7 (−2.1 to 3.5)
Guam	11.7 (9.1 to 15.0)	9.7 (7.7 to 12.2)	8.3 (4.8 to 14.0)	12.7 (7.8 to 19.9)	4.4 (−5.4 to 14.2)
Indonesia	2.1 (1.5 to 2.9)	1.0 (0.7 to 1.6)	2.1 (1.6 to 2.9)	1.1 (0.8 to 1.7)	−1.0 (−1.8 to −0.2)^b^
Iraq	3.6 (2.3 to 5.6)	1.8 (1.0 to 3.4)	3.5 (2.4 to 5.2)	1.9 (1.1 to 3.4)	−1.6 (−3.3 to 0.1)
Kyrgyzstan	5.1 (3.6 to 7.2)	2.4 (1.7 to 3.5)	5.4 (3.8 to 7.7)	2.3 (1.6 to 3.3)	−3.2 (−5.2 to −1.1)^b^
Latvia	3.1 (2.4 to 4.0)	5.3 (4.0 to 7.0)	3.2 (2.4 to 4.1)	5.3 (4.0 to 6.9)	2.1 (0.5 to 3.7)^b^
Lithuania	2.2 (1.6 to 2.9)	3.0 (1.9 to 4.6)	2.0 (1.5 to 2.8)	3.3 (1.8 to 6.1)	1.3 (−1.0 to 3.6)
Mongolia	9.6 (8.1 to 11.2)	8.2 (6.5 to 10.3)	9.6 (8.2 to 11.2)	8.1 (6.5 to 10.0)	−1.6 (−3.8 to 0.7)
Montenegro	1.5 (0.9 to 2.3)	2.2 (1.8 to 2.8)	1.4 (0.9 to 2.1)	2.2 (1.7 to 2.8)	0.8 (0.0 to 1.5)^b^
Nicaragua	4.2 (3.4 to 5.1)	3.4 (2.8 to 4.1)	3.9 (3.1 to 4.8)	3.6 (3.0 to 4.3)	−0.3 (−1.4 to 0.7)
Paraguay	1.9 (1.1 to 3.2)	1.6 (1.0 to 2.7)	1.8 (1.1 to 3.1)	1.6 (1.0 to 2.6)	−0.2 (−1.5 to 1.0)
Peru	1.6 (1.0 to 2.5)	1.9 (1.4 to 2.6)	1.5 (1.0 to 2.4)	2.0 (1.5 to 2.6)	0.5 (−0.4 to 1.3)
San Marino	0.4 (0.1 to 1.6)	0.6 (0.2 to 1.7)	—c	—c	—c
Tajikistan	1.8 (1.2 to 2.6)	1.7 (0.9 to 3.4)	2.1 (1.5 to 3.0)	2.4 (1.4 to 4.0)	0.2 (−1.3 to 1.8)
Uruguay	3.5 (2.8 to 4.4)	1.7 (1.2 to 2.5)	3.6 (2.9 to 4.6)	1.7 (1.1 to 2.5)	−1.9 (−3.1 to −0.8)^b^
Albania	1.9 (1.5 to 2.5)	2.5 (1.9 to 3.4)	1.8 (1.4 to 2.4)	2.5 (1.8 to 3.6)	0.7 (−0.4 to 1.8)
Philippines	2.5 (1.6 to 3.8)	3.0 (1.8 to 5.0)	2.3 (1.5 to 3.5)	3.1 (1.8 to 5.2)	0.8 (−1.1 to 2.7)

Abbreviations: NA, not applicable; pct pts, percentage points.

a Adjusted for sex, age, grade, survey year, and presence of a person who smokes in the household.

b Significant at *P* < .05 in multivariable logistic regression model.

c Value suppressed because the unweighted sample size was less than 35.

### Current any tobacco product use

Change in the prevalence of any tobacco product use among youth are reported in Table 4. Tajikistan had the lowest adjusted prevalence for both the base and latest wave at 0.6% (95% CI, 0.4% to 1.2%) and 0.7% (95% CI, 0.3% to 1.5%), respectively. Similarly, Palau had the highest adjusted prevalence at base wave and the latest wave at 39.3% (95% CI, 36.5% to 42.1%) and 42.3% (95% CI, 37.9% to 46.8%), respectively. Among youth who reported smoking cigarettes or tobacco products other than cigarettes or use of smokeless tobacco on 1 or more days during the past 30 days, 5 sites had a significant decrease in prevalence between waves: Panama, Bhutan, Micronesia, Togo, and San Marino. Conversely, the change in prevalence between waves increased significantly for 3 sites: Albania, Moldova, and Paraguay. The remaining 26 (79%) sites had no significant change in the prevalence between waves for any tobacco product use.

**Table 4:** Change in Prevalence of Any Tobacco Use, Excluding Electronic Cigarettes, Among Youth Aged 13–15 Years, by Country, Global Youth Tobacco Survey, 2012–2020

Country or site	Crude prevalence		Adjusted prevalence^a^		Adjusted prevalence difference, pct pts (95% CI)
	Base wave, % (95% CI)	Latest wave, % (95% CI)	Base wave, % (95% CI)	Latest wave, % (95% CI)	
Argentina	22.4 (19.4 to 25.8)	20.1 (12.4 to 30.7)	19.7 (17.1 to 22.6)	22.3 (14.6 to 32.5)	2.6 (−6.3 to 11.5)
Panama	9.2 (8.3 to 10.3)	8.0 (5.8 to 8.9)	9.3 (8.3 to 10.5)	7.1 (5.8 to 8.7)	−2.2 (−4.1 to −0.4)^b^
Bhutan	28.6 (24.7 to 32.9)	22.2 (20.2 to 24.5)	30.2 (26.0 to 34.7)	22.1 (20.1 to 24.1)	−8.1 (−12.9 to −3.4)^b^
Bosnia and Herzegovina	22.3 (19.5 to 25.3)	24.3 (21.6 to 27.3)	21.4 (18.5 to 24.6)	25.5 (21.6 to 29.7)	4.1 (−1.6 to 9.7)
Brunei	10.6 (6.8 to 16.2)	9.2 (7.1 to 12.0)	10.6 (7.4 to 14.9)	9.1 (7.0 to 11.7)	1.5 (−5.5 to 2.5)
Gaza Strip/ West Bank	15.0 (11.3 to 19.7)	16.2 (11.6 to 22.1)	14.6 (11.8 to 17.8)	15.6 (11.9 to 20.4)	1.1 (−3.2 to 5.3)
Micronesia	38.7 (38.7 to 38.7)	28.9 (26.9 to 31.0)	37.0 (35.6 to 38.5)	29.8 (27.4 to 32.3)	−7.2 (−9.7 to −4.7)^b^
Moldova	9.5 (7.4 to 12.1)	14.6 (12.5 to 17.1)	9.6 (7.5 to 12.2)	14.3 (12.2 to 16.6)	4.7 (1.5 to 7.8)^b^
Palau	41.2 (41.2 to 41.2)	40.0 (35.7 to 44.4)	39.3 (36.5 to 42.1)	42.3 (37.9 to 46.8)	3.0 (−1.5 to 7.5)
Qatar	14.1 (10.8 to 18.2)	12.1 (9.9 to 14.7)	14.0 (11.6 to 16.9)	11.8 (9.8 to 14.1)	−2.3 (−5.0 to 0.5)
Republic of Srpska	10.1 (7.7 to 13.1)	11.8 (9.6 to 14.6)	9.8 (7.7 to 12.4)	12.3 (9.0 to 16.5)	2.5 (−2.0 to 6.9)
Romania	10.6 (8.8 to 12.7)	12.9 (9.5 to 17.2)	9.9 (8.3 to 11.8)	7.9 (6.7 to 9.3)	−2.0 (−4.1 to 0.1)
Senegal	9.7 (6.6 to 14.1)	9.0 (7.6 to 10.7)	8.7 (6.2 to 12.2)	10.3 (8.6 to 12.2)	1.5 (−1.4 to 4.5)
Serbia	15.2 (12.6 to 18.2)	15.6 (15.6 to 15.6)	15.1 (12.5 to 18.0)	16.2 (14.7 to 17.7)	1.1 (−1.2 to 3.5)
Timor−Leste	28.7 (23.0 to 35.2)	30.1 (25.6 to 35.1)	28.5 (23.8 to 33.7)	30.2 (25.8 to 35.0)	1.7 (−4.6 to 7.9)
Togo	8.0 (6.4 to 9.9)	4.0 (2.9 to 5.4)	7.1 (5.7 to 8.7)	4.4 (3.3 to 5.9)	−2.7 (−4.6 to −0.7)^b^
Georgia	11.2 (8.4 to 14.7)	13.9 (11.2 to 17.1)	10.9 (8.1 to 14.4)	14.1 (11.7 to 16.8)	3.2 (−0.7 to 7.1)
Guam	25.9 (22.1 to 30.2)	19.3 (16.6 to 22.3)	21.7 (19.4 to 24.1)	21.7 (19.4 to 24.1)	0 (NA)
Indonesia	19.5 (15.3 to 24.4)	19.2 (17.0 to 21.6)	19.3 (15.8 to 23.3)	19.3 (17.2 to 21.6)	0.0 (−3.7 to 3.7)
Iraq	12.5 (8.8 to 17.6)	15.7 (11.4 to 21.2)	12.7 (9.7 to 16.5)	15.0 (11.4 to 19.5)	2.3 (−1.7 to 6.4)
Italy^c^	23.5 (20.8 to 26.8)	19.8 (17.1 to 22.9)	20.3 (17.0 to 24.2)	19.7 (17.0 to 22.7)	−0.7 (−4.4 to 3.1)
Kyrgyzstan	7.6 (6.0 to 9.5)	6.0 (4.5 to 7.9)	7.9 (6.1 to 10.0)	5.7 (4.3 to 7.4)	−2.2 (−4.6 to 0.3)
Latvia	23.4 (21.8 to 25.9)	23.0 (20.4 to 25.8)	22.9 (21.4 to 24.3)	23.5 (21.1 to 26.0)	0.6 (−2.1 to 3.3)
Lithuania	25.7 (22.7 to 29.0)	22.4 (19.7 to 25.4)	25.3 (22.5 to 28.4)	21.5 (18.2 to 24.6)	−4.1 (−8.9 to 0.6)
Mongolia	13.2 (11.6 to 15.0)	14.0 (12.0 to 16.2)	13.3 (11.7 to 15.0)	13.6 (11.9 to 15.6)	0.3 (−2.1 to 2.7)
Montenegro	8.7 (5.0 to 14.9)	9.8 (8.2 to 11.7)	8.1 (5.0 to 12.9)	10.3 (8.2 to 12.7)	2.2 (−1.0 to 5.3)
Nicaragua	16.0 (14.3 to 17.9)	14.0 (12.5 to 15.7)	10.7 (9.2 to 12.5)	11.5 (10.0 to 13.2)	0.7 (−1.5 to 3.0)
Paraguay	6.6 (5.3 to 8.2)	8.0 (6.9 to 9.4)	6.3 (5.1 to 7.8)	8.2 (7.0 to 9.5)	1.8 (0.1 to 3.6)^b^
Peru	9.1 (6.5 to 12.5)	7.2 (5.6 to 9.1)	8.6 (6.5 to 11.4)	7.4 (5.8 to 9.4)	−1.2 (−3.9 to 1.5)
San Marino	14.4 (11.0 to 18.6)	7.4 (5.3 to 10.3)	14.3 (11.1 to 18.4)	7.4 (5.4 to 10.1)	−7.0 (−11.2 to −2.7)^b^
Tajikistan	3.4 (2.6 to 4.5)	3.0 (2.0 to 4.4)	0.6 (0.4 to 1.2)	0.7 (0.3 to 1.5)	0.0 (−0.6 to 0.7)
Uruguay	11.8 (10.1 to 13.6)	11.5 (7.8 to 16.7)	12.1 (10.5 to 14.0)	11.1 (7.5 to 16.2)	−1.0 (−5.7 to 3.7)
Albania	9.7 (8.3 to 11.4)	15.1 (13.1 to 17.3)	9.7 (8.3 to 11.4)	14.3 (12.5 to 16.4)	4.6 (2.1 to 7.2)^b^
Philippines	14.4 (11.8 to 17.5)	12.4 (10.0 to 15.3)	13.5 (11.1 to 16.3)	13.0 (10.6 to 15.9)	−0.5 (−4.1 to 3.1)

Abbreviations: e-cigarette, electronic cigarette; NA, not applicable; pct pts, percentage points.

a Adjusted for sex, age, grade, survey year, and presence of a person who smokes in the household.

b Significant at *P* < .05 in multivariable logistic regression model.

c Includes cigarette use only; other tobacco products were not included in the country’s survey questionnaire.

### Current electronic cigarette use

In this study, 7 of 10 sites with 2 waves of data indicated a significant increase in e-cigarette use (Table 5). Sites showing increases were Georgia, Italy, Latvia, Albania, Nicaragua, Paraguay, and Peru. Iraq had a significant decrease in e-cigarette use among students, while San Marino and Romania had no significant change in e-cigarette use between waves.

**Table 5:** Change in Prevalence of E-Cigarette Use Among Youth Aged 13–15 Years, Global Youth Tobacco Survey, 2012–2020

Country or site	Crude prevalence		Adjusted prevalence^a^		Adjusted prevalence difference, pct pts (95% CI)
	Base wave, % (95% CI)	Latest wave, % (95% CI)	Base wave, % (95% CI)	Latest wave, % (95% CI)	
Romania	6.7 (5.5 to 8.2)	8.1 (6.9 to 9.5)	7.2 (5.9 to 6.3)	7.4 (6.3 to 8.7)	0.2 (−1.7 to 2.1)
Georgia	5.7 (3.7 to 8.7)	12.6 (9.7 to 16.2)	5.7 (3.7 to 8.7)	12.2 (9.6 to 15.5)	6.6 (2.7 to 10.5)^b^
Iraq	11.0 (8.8 to 13.7)	7.5 (4.3 to 12.7)	10.8 (8.7 to 13.3)	7.1 (4.4 to 11.3)	−3.7 (−6.6 to −0.8)^b^
Italy	8.5 (6.3 to 11.2)	17.5 (14.6 to 21.0)	7.8 (5.8 to 10.5)	17.3 (14.6 to 20.5)	9.5 (6.0 to 13.0)^b^
Latvia	9.9 (8.1 to 12.1)	18.0 (16.4 to 19.7)	9.7 (8.1 to 11.7)	18.3 (16.9 to 19.9)	8.6 (6.4 to 10.9)^b^
Nicaragua	5.3 (4.1 to 7.0)	8.6 (7.3 to 10.1)	5.2 (4.0 to 6.8)	8.5 (7.3 to 10.0)	3.3 (1.4 to 5.3)^b^
Paraguay	3.7 (2.6 to 5.1)	12.6 (11.0 to 14.3)	3.6 (2.6 to 5.0)	12.5 (10.9 to 14.1)	8.8 (6.9 to 10.8)^b^
Peru	2.4 (1.6 to 3.4)	7.2 (5.6 to 9.1)	2.2 (1.5 to 3.2)	6.6 (5.5 to 8.0)	4.4 (3.0 to 5.8)^b^
San Marino	5.9 (3.9 to 8.9)	8.9 (5.9 to 13.0)	5.8 (3.8 to 8.8)	9.1 (6.3 to 13.0)	3.3 (−0.8 to 7.5)
Albania	9.7 (8.3 to 11.4)	9.2 (7.8 to 10.8)	5.5 (4.6 to 6.5)	9.2 (7.7 to 10.9)	3.7 (1.7 to 5.7)^b^

Abbreviations: e-cigarette, electronic cigarette; pct pts, percentage points.

a Adjusted for sex, age, grade, survey year, and presence of a person who smokes in the household.

b Significant at *P* < .05 in multivariable logistic regression model.

## Discussion

Based on 2012–2020 GYTS data from 34 sites, we found that tobacco product use among students who reported currently using various products on 1 or more days remained unchanged in most sites. However, we found that self-reported e-cigarette use among students increased in 7 of 10 sites. It is important to note that our definition for “tobacco product” excluded e-cigarettes, which differs from how CDC defines it in the US (12).

The findings from our analysis regarding cigarette smoking and the use of other tobacco products differ from those reported in another study that used GYTS data (5). Ma et al found that cigarette smoking decreased from 1999 to 2018 in the majority of sites included in their analysis, while the prevalence of using tobacco products other than cigarettes increased in most sites. However, it should be noted that Ma et al included 143 sites that conducted 2 or more waves of GYTS between 1999 and 2018. Our analysis focused on sites that conducted 2 or more waves since CDC and WHO implemented the updated GYTS standard protocol in 2012; this was done to ensure consistency with the core questions sites included in their survey. Moreover, our analysis included data from sites that implemented GYTS in 2019 and 2020.

Many countries that implemented WHO FCTC reported significant declines in tobacco product use among youth (5,13). However, our findings show that progress in reducing tobacco demand among youth has stalled. Our analysis also found increased use of e-cigarettes — especially in the European region — consistent with previous reports (14,15). Although few sites in this analysis reported comparison data for e-cigarettes, their increased use might be indicative of dual tobacco and e-cigarette use, or a shift from cigarette to e-cigarette use. The transition from cigarette smoking to e-cigarette use has been reported in the US, where e-cigarettes are now the most popular tobacco product used by middle and high school students (12). The rapidly changing landscape of commercial tobacco poses a challenge for many governments because it is often more difficult to develop regulations at the same speed that new products are introduced and marketed to youth by the tobacco industry (16). Many of these new products include flavors that youth find appealing, such as fruit, menthol or mint, dessert, and alcohol; the use of flavors is known to mask the harshness of nicotine, thus making the product more palatable and pleasurable (3). The increased use of e-cigarettes in the sites included in this analysis is especially concerning, given that youth who use high-concentration nicotine solutions have an increased likelihood of progressing to higher frequency and intensity levels of e-cigarette use (vaping) and smoking (17).

The stall in reducing tobacco product use among youth in most of the sites analyzed, along with the increased use of e-cigarettes, further highlights why policy makers might want to consider implementing and enforcing cost-effective, evidence-based interventions and strategies aimed at reducing tobacco demand. Restrictions on direct and indirect advertising, promotion, and sponsorship and increased tobacco excise taxes have proven effective in reducing tobacco use among youth (18,19). Globally, few Parties to the WHO FCTC have adopted all MPOWER demand reduction measures — a comprehensive package of 6 cost-effective measures that help countries reduce tobacco demand (7) — at the best-practices level. Of the sites included in our analysis with available data from WHO (n = 32), most (84%) adopted less than 4 MPOWER measures at the best-practice level as of 2020; none adopted all 6 measures at the best-practice level (3). Although global monitoring of e-cigarette use among youth has improved in recent years, continuous monitoring of the uptake of all electronic nicotine delivery systems, flavored tobacco products, and other emerging products (eg, synthetic nicotine products) is paramount to ensure evidence-based interventions are adopted and implemented to protect youth.

A strength of our study is that we used the latest nationally representative GYTS data, making this report the most up-to-date evidence on tobacco product use among youth globally. Additionally, our analysis included only data from sites that implemented GYTS after the standard protocol was updated in 2012, thus ensuring consistency in tobacco product use definitions across all sites. Our study also has limitations. First, GYTS collects self-reported data, which are subject to recall and social desirability bias; however, it should be noted that GYTS seeks to minimize social desirability bias by maintaining anonymity during data collection. Second, because we only included GYTS data collected after 2012, we could not assess longer-term trends because the GYTS methodology changed slightly in 2012. Also, the years of data included varied by country. Third, our findings are generalizable to students enrolled in formal schooling (ie, public and private schools); they are not generalizable to students who accessed schooling through informal channels or those not enrolled in school. Fourth, although we controlled for survey year in our analysis, we did not account for various policies and programs that could have been implemented by sites between survey waves. Lastly, the phrasing of the question regarding tobacco products other than cigarettes limits our ability to discern whether the use of specific combustible products (eg, shisha/water pipes, cigars, bidis) increased, decreased, or remained unchanged during the period examined.

In conclusion, our findings indicate that among most of the 34 sites that implemented 2 or more waves of GYTS during 2012–2020, tobacco product use remained unchanged. The stall in progress likely indicates slow or limited adoption, implementation, and enforcement of comprehensive tobacco control policies, such as those outlined in the WHO MPOWER package. Furthermore, the stall in progress might also be influenced by aggressive marketing of tobacco products to youth — particularly on social media (20,21) — and the increased affordability of tobacco products in sites that have seen drastic economic growth in the last decade. The finding that e-cigarette use increased among youth highlights the importance of regular monitoring to better understand its public health impact. Furthermore, with some countries classifying e-cigarettes as a cessation device for adults, regular monitoring of e-cigarettes among youth might provide evidence for policy makers to consider regulations that balance potential cessation benefits for adults against potential harms for youth and other vulnerable populations, including pregnant women. Overall, continued surveillance and monitoring of tobacco and nicotine products are critical to strengthening tobacco control policies and strategies at the national, regional, and international levels.
